# Gamma photon attenuation measurement as a technique for monitoring liquid composition

**DOI:** 10.1155/S1463924684000171

**Published:** 1984

**Authors:** Sandra Everett, David J. Malcolme-Lawes

**Affiliations:** Chemistry Department King's College London Strand London WC2 UK


Journal of Automatic Chemistry, Volume 6, Number 2 (April–June 1984), pages 84–87

### Gamma photon attenuation measurement as a technique for monitoring liquid composition

**Sandra Everett and David J. Malcolme-Lawes**
*Chemistry Department, King's College London, Strand, London WC2*

### Introduction

Many industrial processes involve large-scale flows of liquid mixtures or solutions in situations where it is desirable for the composition of the flow to be monitored. Such monitoring can be achieved through batch sampling or by using some property of the liquid system, such as its pH or its optical density, to provide a continuous measurement related to liquid composition. Batch sampling complicates a monitoring procedure which might otherwise be suitable for a high degree of automation, and in any event is likely to be expensive. However, many continuous monitoring techniques require the presence of delicate special devices (such as electrochemical probes) or windows (such as would be required for UV absorption measurements) in contact with the flowing liquid system. Furthermore, continuous monitoring techniques which rely on a specific property of the liquid (such as its optical density at a specified wavelength) lack versatility and may be rendered unsuitable for a particular application by a relatively small change in the liquid system, such as the addition of an impurity.

A physical property which provides a finite and measurable characteristic for any material is its linear attenuation coefficient for X-ray or gamma photons [1 and 2]. Such photons can be obtained at highly predictable rates from the decay of a variety of radionuclides [3]. Some isotopes emit gamma photons directly (for example nickel-60, the daughter of cobalt-60), while others may emit X-rays after electron capture decay. X- and gamma photons are generally emitted at discrete energies, and some radionuclides may be used as sources of monoenergetic photons. All beta-emitting isotopes can be used to generate Bremsstrahlung radiation, by allowing the beta particles to be stopped by a heavy target. Bremsstrahlung photons have a continuous energy spectrum up to a maximum energy (approximately that of the incident beta particles) and, because of the inverse wavelength dependence of the intensity spectrum, may provide a useful source of low-energy photons.

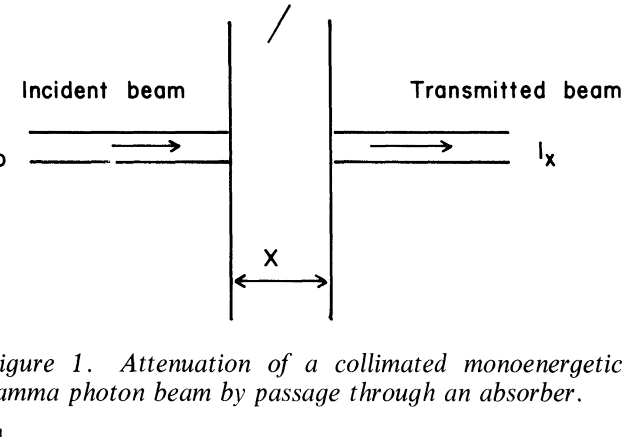

*Figure 1. Attenuation of a collimated monoenergetic gamma photon beam by passage through an absorber.*

When energetic photons pass through matter they become scattered and absorbed [2], principally by photoelectric and Compton processes at the energies of interest here (< 1 MeV). As illustrated in figure 1, the intensity of a collimated beam of monoenergetic photons, with an initial intensity $I_0$ is reduced to $I_x$ after travelling a distance $x$ through matter, where, according to the Bouger-Lambert-Beer law:

$$I_x = I_0 \exp(-ux) \qquad (1)$$

and $u$ is the linear absorption coefficient of the matter concerned. $u$ is a quantity which depends on the energy of the photons in the beam and on the probability of interaction between those photons and the molecules which constitute the matter through which they pass.

For a mixture of materials the linear absorption coefficient is given by:

$$u = p \sum_i g_i (u/p)_i \qquad (2)$$

where $p$ is the density of the mixture, $g_i$ is the mass fraction of component $i$, $(u/p)_i$ is the mass absorption coefficient of component $i$, and the summation is over all components of the mixture. The mass absorption coefficient is the quantity which is related to the molecular properties of the component in question, being given by:

$$(u/p)_i = o_{total}(N/M) \qquad (3)$$

where $N$ is the Avagadro constant, $M$ is the molecular weight of the material, and $o_{total}$ is the total cross-section for the removal of photons from a monoenergetic collimated beam.

Equation (2) may also be used to calculate the mass absorption coefficients for compounds from the $(u/p)$ of their constituent atoms, these in turn being calculated from equation (3) using tabulated or theoretical values for the atomic scattering cross-sections [4].

Thus for any liquid mixture and any photon energy it is possible to estimate the linear absorption coefficient, given that the density can be estimated with reasonable accuracy and that the necessary atomic scattering cross-sections are available. A few such calculations soon indicate that the attenuation $(I_x/I_0)$ of a beam of gamma photons can be used to provide a moderately sensitive means of monitoring mixture composition, and several applications of this technique to solid systems have been reported [5–8].

This paper introduces an instrument for monitoring the attenuation of a collimated beam of gamma photons by a liquid mixture flowing through a pipe. The apparatus and some preliminary results obtained with trial mixtures are discussed below. However, the sensitivity of the technique is dictated both by the difference in $u$ for the materials which make up the mixture, and by the precision with which $I_x/I_0$ may be monitored. For this reason we first consider the fundamental requirements for the measurement of the intensity of a gamma photon beam.

### Accuracy in gamma monitoring

In principle, the intensity of a beam of photons can be measured to any desired degree of accuracy, but in practice there are some limitations on what may be achieved. Gamma photons with energies above about 25 keV may readily be detected by observing their interaction with a fluorescent material and measuring the intensity of the UV/visible photons produced. One could use plastic scintillator materials, which fluoresce very rapidly (*c*. 1 ns) after excitation, but which have rather poor stopping powers for gamma photons. Alternatively, inorganic crystal scintillators (such as thallium-activated sodium iodide) may be used, providing good stopping powers, but with fluorescent lifetimes as long as a microsecond. The stopping power is important because it dictates the size of the detector required for a given efficiency, and consequently the background level recorded from natural radioactivity and cosmic radiation. The fluorescent lifetime of the detector material is important because it dictates the maximum gamma detection rate which can be recorded by counting the individual scintillations (as opposed to integrating the total light emission from the scintillator).

The difficulty in detecting gamma photons by integrating the total light emission from a scintillator arises because one loses the ability to discriminate against scintillations which arise from sources other than the detection of a gamma photon of the desired energy. For this reason the counting of individual scintillations corresponding to gamma photons within a predetermined energy range was chosen as the most suitable technique for monitoring beam intensity without interference from photons which had been scattered by the pipework carrying the flowing liquid. Sodium iodide scintillation crystals were chosen for the detectors because they are highly efficient in the energy range of interest (25–250 keV), and relatively compact detector assemblies could be used. The choice of sodium iodide imposed a limitation of about $10^5\,s^{-1}$ as the maximum gamma detection rate which could be realised without errors caused by pulse overlap becoming important.

The technique adopted for the measurement of the beam intensity involved counting pulses from the scintillation detector for a preset time period, the beam intensity then being taken as proportional to the count rate. If the number of counts recorded in a time interval $t$ is $N$, then the intensity may be written:

$$I \pm \sigma_I = k(N \pm \sigma_N)/(t \pm \sigma_t)$$

where the $\sigma_i$ are the errors associated with each quantity and $k$ is a constant. $\sigma_N$ is the variance of the count and, assuming a Poisson distribution for the counts, is given by $N^{1/2}$ [9]. $\sigma_t$ may be determined by measurements on the timing system used—in our case $\sigma_t$ is about $10^{-7}$ s, and this is small enough to be neglected for all values of $t$ used in the present work. Consequently:

$$I \pm \sigma_I = kN/t \pm kN^{1/2}/t.$$

The fractional error in the intensity measurement is therefore:

$$\sigma_I/I = N^{1/2}.$$

If the approximate pulse counting rate is $R$ s$^{-1}$, and if $R < 10^5\,s^{-1}$, then:

$$\sigma_I = (Rt)^{1/2}.$$

For our preliminary experiments we aimed for a value of $R$ of approximately $10^4\,s^{-1}$, so that the error in our intensity measurements was:

$$\sigma_I/I = 10^{-2}\,t^{-1/2}$$

or about 1% for a counting time of 1 s, 0·1% for a counting time of 100 s. These values could be improved by using a larger value of $R$, although the restriction imposed by the use of sodium iodide detectors does place a limit on the improvement possible. Further improvement would necessitate the use of faster scintillator materials.

### Experimental

The apparatus used for monitoring the absorption of gamma photons by flowing liquids is shown schematically in figure 2. The radioactive source was a 14 mCi 'point source' of Americium-241 housed in a 10 mm × 3 mm diameter stainless-steel holder (Amersham International PLC, code number AMC.24). The source was positioned in the centre of a 50 mm × 30 mm diameter lead pot, and held in position by a softwood support. A 3 mm diameter hole in the top of the pot allowed the exit of a semi-collimated beam of gamma photons, with a convergence angle of approximately 20°.

The resulting beam consists largely of 60 keV 241 Am gamma photons with a smaller (*c*. 20%) component of 25 keV photons (which were discriminated against electronically) and produces a count rate from the detector of approximately $4 \times 10^4\,s^{-1}$ in the absence of a sample. The beam was allowed to pass normally through a flow cell of 10 cm diameter glass tubing (2·5 mm wall thickness) through which sample liquid was pumped at a flow rate of approximately $5\,l\,min^{-1}$. The attenuated beam was then monitored using a NaI(Tl) scintillation detector probe (type 5.42, Mini Instruments Ltd) connected to a 24 bit pulse counter, which was read at intervals by a PET microcomputer. A second 24 bit pulse counter was used to count pulses from a 100 kHz clock oscillator. Latching of the 24 bit interfaces and the resetting of both counters to zero was accomplished using 1 ms wide pulses synchronized with the leading edge of the clock oscillator, resulting in time reproducibilities of better than $10^{-7}$ s. The microcomputer evaluated the count rate for either a preset counting period or for predetermined statistical error limits on the record count.

Samples consisted of total volumes of approximately 3 l, recirculated through the flow cell using a centrifugal pump.

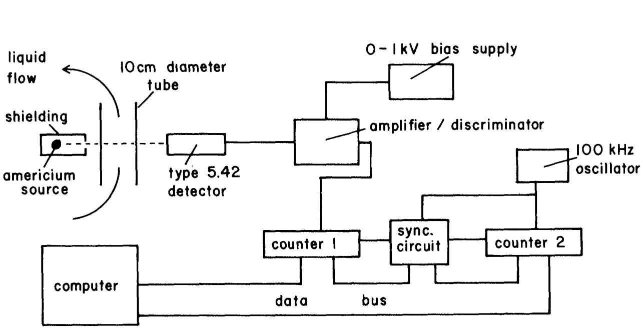

*Figure 2. Schematic representation of experimental system used for monitoring the intensity of 60 keV gamma photon beam after passage through liquid mixture.*

Standard laboratory-grade reagents were used throughout, and mixtures and solutions were made up to an estimated accuracy of 1%. Results consisting of measurements of photon transmission, $100 \times I/I_0$, were obtained for binary liquid mixtures, both homogeneous (ethanol/water) and heterogeneous (decane/water), and for aqueous solutions, ionic (sodium chloride) and non-ionic (sucrose).

### Results and discussion

The measured photon transmissions as functions of sample composition are shown in figures 3–6. In each case a theoretical plot is also included, although the absolute position of such curves is subject to relatively large errors, probably as a result of the use of the theoretical atomic absorption coefficient for hydrogen (in reality this quantity is significantly influenced by chemical bonding). The atomic attenuation coefficients used to derive the theoretical linear attenuation coefficients are collected in table 1.

*Table 1. Attenuation coefficients.*

(a) Atomic attenuation coefficients

| Element | u/p/cm$^2$g$^{-1}$ |
|---|---|
| H | 0·3260 |
| C | 0·1751 |
| O | 0·1903 |
| Na | 0·2310 |
| Cl | 0·4044 |

(b) Linear attenuation coefficients derived for compounds

| Sample | u/cm$^{-1}$ |
|---|---|
| Water | 0·2054 |
| Decane | 0·1449 |
| Ethanol | 0·1579 |
| 0·5 M sucrose | 0·2208 |
| 0·5 M NaCl | 0·2161 |

Figure 3 shows the results for two completely miscible liquids, ethanol and water, and figure 4 the results for aqueous solutions of a non-dissociating solute, sucrose, over the concentration range 0–2 M. In both cases the error bars shown are the (theoretical) statistical errors for a counting time of 10 s. The magnitude of the error bars is such that the error in a composition estimate would be about 4% of the %$H_2O$ value in the case of ethanol/water mixtures, and about 0·2 M in the case of the sucrose solutions.

Figure 5 shows the results obtained for aqueous solutions of sodium chloride, where a relatively sensitive variation is observed, owing to the large mean atomic number difference between solvent and solute. Figure 6 contains the results obtained for decane/water mixtures, although in this case the experimental results are also subject to errors which arose through the difficulty we experienced in maintaining a constant composition flow at certain compositions using our relatively crude mixing and pumping system.

In all cases the error bars are those which would apply to single counts of 10 s duration, although the curves are drawn through the smaller error ranges which result from 100 s counting times. The smoothness of the curves indicates that a dramatic improvement is obtained with the order of magnitude increase in counting time, and this same improvement is also to be expected from an order of magnitude increase in the activity of the photon source. A 10-fold increase in source activity would

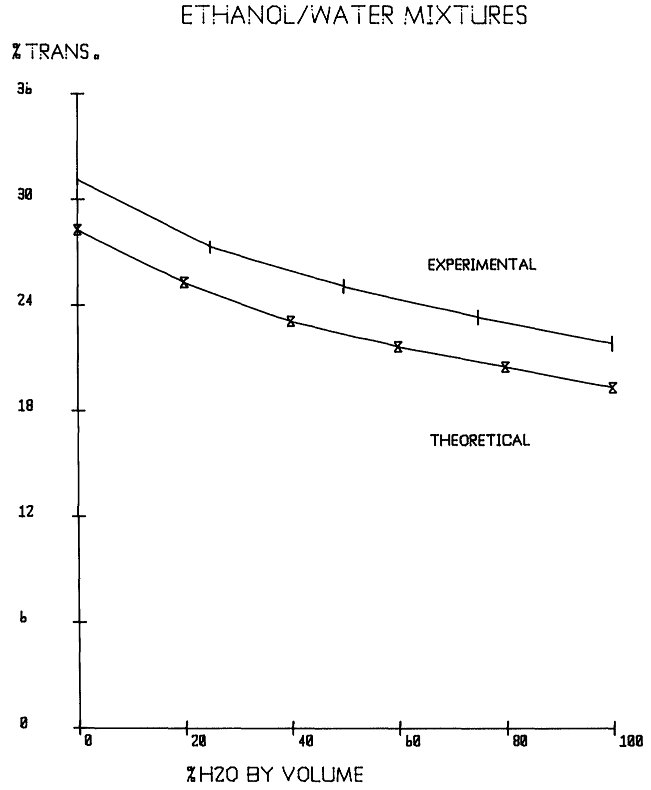

*Figure 3. Gamma transmission as a function of mixture composition for ethanol/water mixtures. Flow rate is approximately 3 l min$^{-1}$ through a 10 cm diameter pipe.*

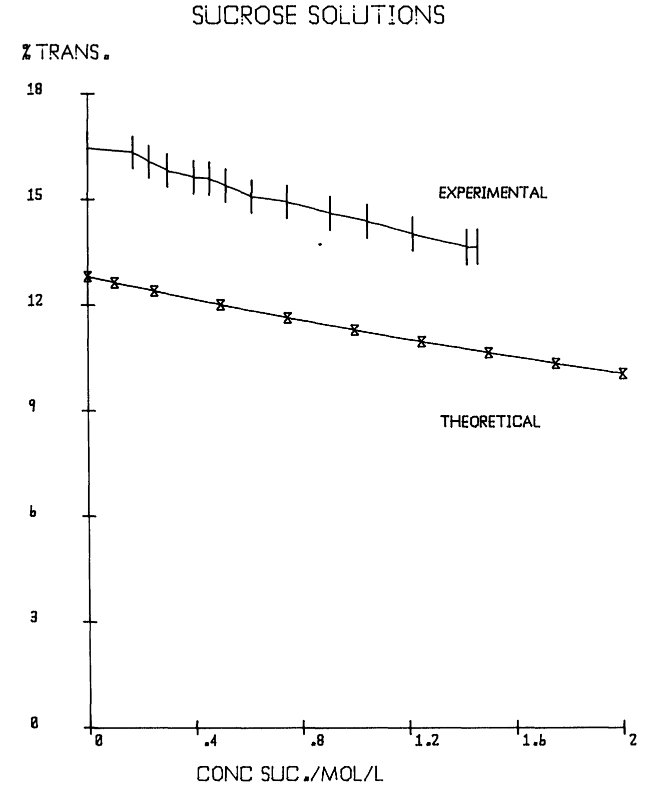

*Figure 4. Gamma transmission as a function of mixture composition for aqueous sucrose solutions. Flow rate is approximately 3 l min$^{-1}$ through a 10 cm diameter pipe.*

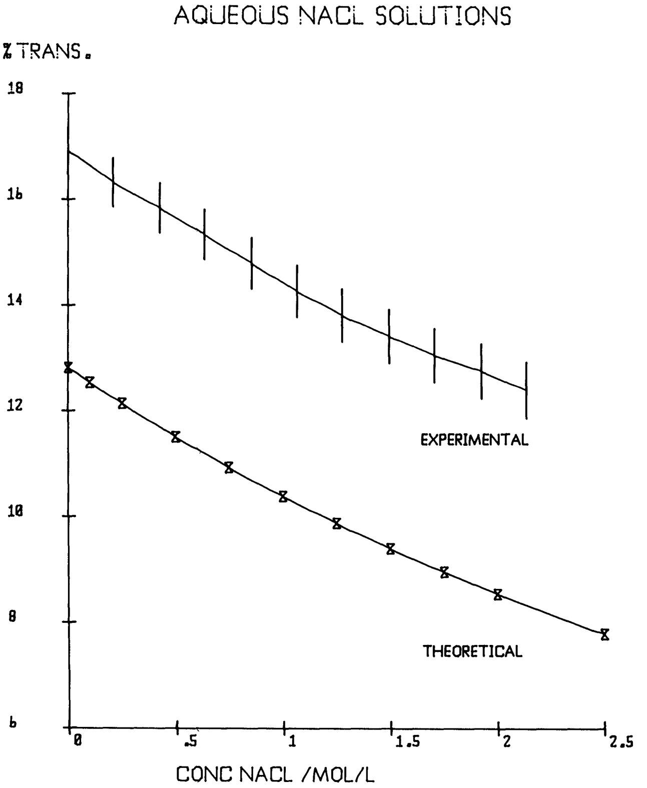

*Figure 5. Gamma transmission as a function of mixture composition for aqueous sodium chloride solutions. Flow rate is approximately 3 l min$^{-1}$ through a 10 cm diameter pipe.*

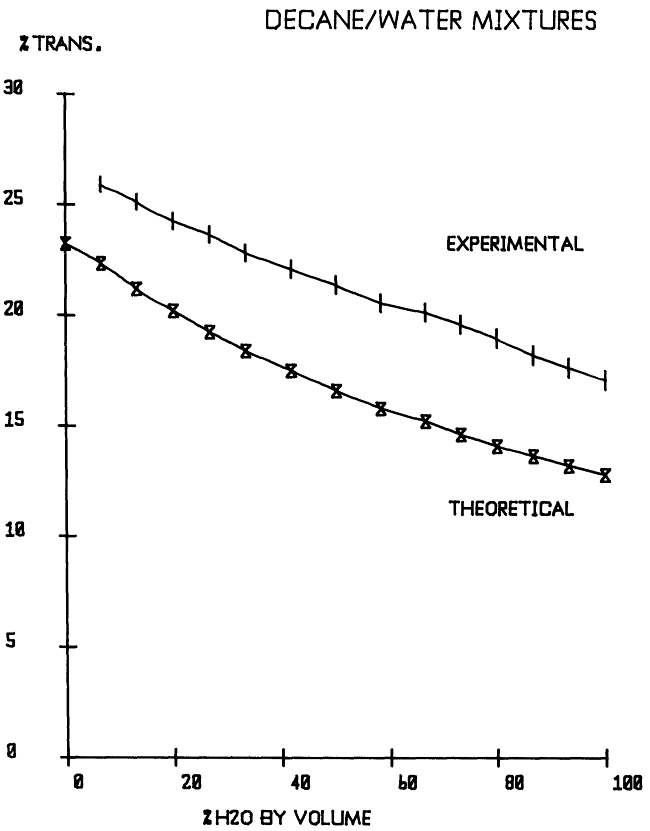

*Figure 6. Gamma transmission as a function of mixture composition for decane/water mixtures. Flow rate is approximately 3 l min$^{-1}$ through a 10 cm diameter pipe.*

have produced the same experimental curves with 10 s counting times and the error bars would have been reduced by a factor of 3·3.

The difference between the experimental and theoretical curves can be accounted for by the inadequacies of the atomic attenuation coefficients, particularly that for hydrogen, and is not particularly important in the present context. It is nevertheless encouraging to note that the shapes and slopes of the theoretical and experimental curves are in much closer agreement. This will allow the use of computer simulation techniques to determine the optimum source energies and path lengths for future experiments. Excellent agreement between the theoretical and experimental curves is readily achieved by the empirical adjustment of one or more of the atomic attenuation coefficients.

These results clearly demonstrate that gamma attenuation may be a useful technique for monitoring the composition of a variety of binary mixtures, in the forms of flowing liquids. A reasonable degree of accuracy may be achieved if an adequate transmission intensity is available, and there is a predictable relationship between the transmission intensity and the time required for measurements of a specified precision.

### Acknowledgement

This work was supported by the BP Sunbury Research Centre.

### References

1. Friedlander, G., Kennedy, J. W. and Miller, J., In *Nuclear and Radiochemistry* (Wiley, New York, 1966).
2. *International Tables for Crystallography*, Volumes II and III (The Kynoch Press, 1968).
3. Lederer, C. M., Hollander, J. M. and Perlman, I., In *Table of Isotopes* (Wiley, New York, 1967).
4. Hubbell, J. H., *Radiation Research*, **70** (1977), 58.
5. Gilson, A., Cohem, M. and Day, F., *AEC Accession 43049, Rept. No. AED-CONF-65-197-3* (1965).
6. Burek, R., Mirkavski, C. and Pawlak, J., *Nukleonika*, **13** (1968), 535.
7. Bodin, F. and Preuss, L., *Proc. ERDA Symp. X- and Gamma Ray Source Applications* (1976), 147.
8. Merkulov, V., and Klimusher, A. Zh., *Fizica Khimica*, **34** (1960), 1371.
9. Malcolme-Lawes, D. J., *Introduction to Radiochemistry* (Macmillan Press, London, 1979), 40.

### BOOKS

The Editor is anxious to establish a book review section in *Journal of Automatic Chemistry*. He would welcome readers who are about to publish a book, or having a paper in a proceedings volume, either asking their publisher to send a review copy to him or letting him know about the publication.

*Information to Dr P. B. Stockwell, P. S. Analytical Ltd, 2 Eagles Drive, Tatsfield, Westerham, Kent TN16 2PB, UK.*

